# Redox Switches in Noise-Induced Cardiovascular and Neuronal Dysregulation

**DOI:** 10.3389/fmolb.2021.784910

**Published:** 2021-11-18

**Authors:** Katie Frenis, Marin Kuntic, Omar Hahad, Maria Teresa Bayo Jimenez, Matthias Oelze, Steffen Daub, Sebastian Steven, Thomas Münzel, Andreas Daiber

**Affiliations:** ^1^ Department of Cardiology, Molecular Cardiology, University Medical Center, Mainz, Germany; ^2^ Boston Children’s Hospital and Harvard Medical School, Boston, MA, United States; ^3^ German Center for Cardiovascular Research (DZHK), Partner Site Rhine-Main, Mainz, Germany

**Keywords:** sources of reactive oxygen species, redox switches, oxidative stress, cardiovascular disease, neuronal complications, traffic noise exposure

## Abstract

Environmental exposures represent a significant health hazard, which cumulatively may be responsible for up to 2/3 of all chronic non-communicable disease and associated mortality (Global Burden of Disease Study and The Lancet Commission on Pollution and Health), which has given rise to a new concept of the exposome: the sum of environmental factors in every individual’s experience. Noise is part of the exposome and is increasingly being investigated as a health risk factor impacting neurological, cardiometabolic, endocrine, and immune health. Beyond the well-characterized effects of high-intensity noise on cochlear damage, noise is relatively well-studied in the cardiovascular field, where evidence is emerging from both human and translational experiments that noise from traffic-related sources could represent a risk factor for hypertension, ischemic heart disease, diabetes, and atherosclerosis. In the present review, we comprehensively discuss the current state of knowledge in the field of noise research. We give a brief survey of the literature documenting experiments in noise exposure in both humans and animals with a focus on cardiovascular disease. We also discuss the mechanisms that have been uncovered in recent years that describe how exposure to noise affects physiological homeostasis, leading to aberrant redox signaling resulting in metabolic and immune consequences, both of which have considerable impact on cardiovascular health. Additionally, we discuss the molecular pathways of redox involvement in the stress responses to noise and how they manifest in disruptions of the circadian rhythm, inflammatory signaling, gut microbiome composition, epigenetic landscape and vessel function.

## Introduction

Around 50% of the world’s population currently resides in urban environments, following a trend of increasing worldwide urbanization which is expected to continue in the near future (The World Bank, 2020). By 2050, the United Nations (UN) estimates that 6.68 billion people will reside in cities (United Nations, 2018). These demographic shifts, alongside the SARS-CoV-2 pandemic pushing the employment paradigm towards a scheme of working from home (Pew Research Center, 2020), make a healthy home environment and healthy urban planning more important than ever (Munzel et al., 2021b). As an important component of the exposome (Wild, 2005), or the cumulation of health-related exposures over the course of life (Vrijheid, 2014; Sainani, 2016; Vineis et al., 2020), excess noise is an increasingly recognized health risk factor to which urban dwellers are particularly susceptible because it is found at potentially hazardous levels in highly trafficked areas and in areas surrounding airports. The effects of noise have been well-quantified in the context of occupational hearing loss, wherein it has been reported that 22 million Americans are exposed to hazardous levels of noise per year (Tak et al., 2009), and several studies have indicated that exposure to levels of noise above 85 decibels (dB) in industrial settings has a correlation with increased systolic blood pressure (Kerns et al., 2018; Li et al., 2019b). These numerous studies do not account for exposures at low or moderate levels outside of the workplace, which are sound pressure levels more relevant to daily exposures. Noise from more common sources has also recently been implicated as harmful as put forward in the most recent World Health Organization (WHO) Noise Guidelines for the European Region (2018) and meta-analysis thereof (Guski et al., 2017; Clark and Paunovic, 2018; Kempen et al., 2018). Based on the latter data, significant health effects have already become evident upon chronic exposure to an average sound pressure level of >45 dB(A) during the night and >55 dB(A) during the day. Traffic noise, particularly during the night, appears to be a major contributor to the noise burden of the average person (Sorensen et al., 2011; Heritier et al., 2019; Munzel et al., 2020).

Traffic noise arises from several sources and can span a wide variety of intensities and frequencies, making the correlation between exposure and effects on human health difficult to fully elucidate. The Caerphilly study was established in 1984 and was conducted until the mid-1990s with the goal of correlating ischemic heart disease with road traffic noise exposure using exposure levels as mapped in 1984. The study did not find a significant association between ischemic heart disease and noise exposure (51–70 dBA, 6–22 h), but did uncover associations between risk factors including increased systolic blood pressure, estradiol, total cholesterol, plasma viscosity, antithrombin III, cortisol, and decreased platelet count (Babisch et al., 1988). These data provided the first notable insights into risk posed to cardiovascular health by noise below the threshold commonly accepted as being hazardous. Since these initial insights, many more population-based studies have been conducted (reviewed in detail in Münzel et al. Munzel et al., 2018a; Munzel et al., 2020; Munzel et al., 2021a). Amongst these were 22 studies yielding high quality evidence linking road traffic noise and incidence, prevalence, or mortality from ischemic heart disease (van Kempen et al., 2002; Kempen et al., 2018). Correlations between traffic noise exposure and other cardiovascular diseases like hypertension, stroke, and diabetes were generally positive but suffered from low quality evidence due to heterogeneity of methods (van Kempen et al., 2002; European Region, 2018). These epidemiological findings are particularly concerning given the near-ubiquitous presence of noise; the WHO has estimated that 40% of Europeans are exposed to road traffic noise exceeding the strongly recommended daytime level of 55 dB(A) and 30% exceed the lower level of 45 dB(A) recommended at night (Fritschi et al., 2011). More densely populated Asian urban centers could exceed even those estimates, with a noise day-evening-night level (L_den_) of 60–65 dB(A) (Lelieveld et al., 2015).

**FIGURE 1:**
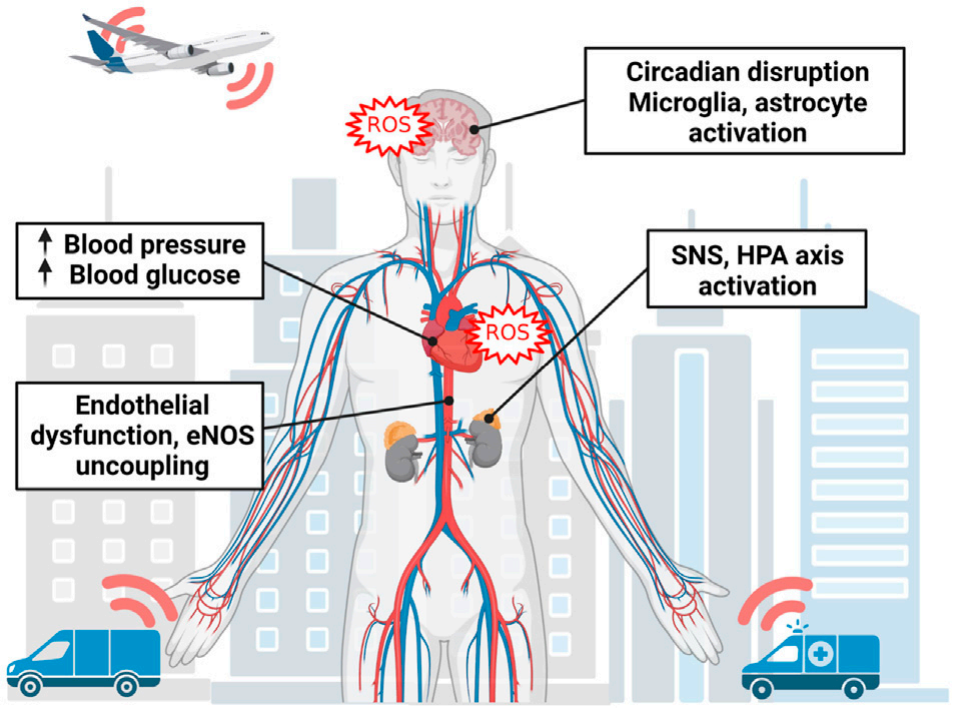
Overall mechanism of noise-triggered adverse health effects. Noise perception starts in the brain leading to neuronal activation in association with disruption of circadian rhythms (especially by nighttime noise causing sleep deprivation and fragmentation), neuroinflammation and cerebral oxidative stress. Noise activates down-stream stress responses such as activation of the sympathetic nervous system (SNS) and the hypothalamic-pituitary-adrenal (HPA) axis leading to stress hormone release such as catecholamines and cortisol with secondary activation of the renin-angiotensin-aldosterone system. This cascade will converge in oxidative stress and inflammation in association with eNOS uncoupling, endothelial dysfunction and high blood pressure as well as hyperglycemia, well-known triggers of cardiovascular sequela. Image was created using Biorender.com.

The epidemiological evidence that associates traffic noise with onset and progression of cardiovascular disease is abundant, but in order to truly understand how noise and cardiovascular disease are connected, deeper mechanistic insights are necessary. In human field studies, vitamin C was shown to alleviate endothelial dysfunction associated with one night of aircraft noise exposure (Schmidt et al., 2013; Herzog et al., 2019). This implies that oxidative stress has an important role in the underlying pathophysiology (Heitzer et al., 2001), which is also compatible with a number of cardiovascular sequela that were associated with noise exposure (Figure 1). It was even shown that aircraft noise exposure for one night increased the serum levels of 3-nitrotyrosine-positive proteins in patients with established coronary artery disease (Schmidt et al., 2015; Kroller-Schon et al., 2018), whereas train noise exposure for one night caused a shift to pro-oxidative and pro-atherothrombotic milieu of the plasma proteome in healthy volunteers (Herzog et al., 2019). These studies (Schmidt et al., 2013; Herzog et al., 2019) and others (Schmidt et al., 2021) have demonstrated that the interruption of sleep may be an important mechanism for prompting this pro-oxidative environment, and in exposure while awake, annoyance in response to noise appears to be correlated to anxiety and depression (Beutel et al., 2016; Beutel et al., 2020) as well as atrial fibrillation (Hahad et al., 2018; Hahad et al., 2021b). These human lines of evidence for a role of oxidative stress or adverse redox signaling for noise-induced adverse (cardiovascular) health effects were further supported by numerous mechanistic animal studies that will be discussed in detail within this review and were already partially summarized previously (Munzel et al., 2018a; Munzel et al., 2018b; Munzel et al., 2020; Munzel et al., 2021a).

## Animal Research on Noise-Induced Cardiovascular and Neuronal Dysregulation

Research into the mechanisms by which noise exerts detrimental impact on human health has been underway for decades, though it has been intermittent. An important paradigm was put forth by Babisch (Babisch, 2002), which stipulates that noise could have both a direct and indirect pathway in its impact on human health. The direct pathway entails auditory damage by high-intensity sound exposure, which culminates in damage of the inner ear and stress responses (Figure 1). The indirect pathway is relevant to “sub-hazardous” noise exposures, including traffic noise, and manifests as annoyance or disturbance of sleep. These cognitive/emotional and physiological responses intersect with the direct pathway by causing stress responses, which can then manifest as cardiometabolic disease (Daiber et al., 2019a). There is overlap between the adverse effects of stress and sleep disruption, which are cardiovascular risk factors in their own right, but can also lead to increases in catecholamine, adrenocorticotropic hormone (ACTH), and cortisol secretion, circadian disruption and decreased melatonin production, decreased insulin sensitivity and leptin levels, increases in ghrelin and appetite, upregulation of inflammatory proteins such as tumor necrosis factor alpha (TNFα), interleukins (e.g., IL1, IL6), and C-reactive protein (CRP), as well as increases in oxidative stress (Medic et al., 2017). Importantly, stress responses are triggered in the brain and activate the sympathetic nervous system (SNS), hypothalamic-pituitary-adrenal (HPA) axis, and endocrine systems which can lead to presentation of the aforementioned cardiovascular risk factors through hormonal signaling (Daiber et al., 2019a).

The noise reaction scheme has been generally upheld by pre-clinical work in the field of noise research (Munzel et al., 2018a; Munzel et al., 2018b; Munzel et al., 2020; Munzel et al., 2021a). Evidence that stress responses are a key component in the appearance and exacerbation of cardiovascular risk factors has been both explicitly and tangentially explored. Enhanced glutaminergic signaling in the amygdala of rats (Singewald et al., 2000) and amygdalar activation in humans (Osborne et al., 2020; Hahad et al., 2021a; Osborne et al., 2021) demonstrates a stress-induced arousal in response to noise. Activation of the HPA axis is evident in the increased plasma corticosterone of noise-exposed rats and increases in plasma cortisol in noise-exposed mice aligns with readouts of sympathetic activation, adrenaline and noradrenaline, increased in plasma and kidney of noise-exposed mice (Munzel et al., 2017) and rats (Gannouni et al., 2013). Activation of these stress response systems accounts for the adverse cardiovascular readouts detected in noise-exposed animals, which includes several reports of increases in blood pressure (Peterson et al., 1981; Peterson et al., 1984a), increased myocardial fibrosis (Herrmann et al., 1994), as well as atrial interstitial fibrosis (Lousinha et al., 2020). Our own work sheds light on the molecular workings behind these effects. Using our standardized noise exposure protocol, we reliably report elevation of blood pressure in noise-exposed animals, which exacerbates pre-existing hypertension. Our model also finds increases in leukocyte infiltration into the aortic endothelium, causing endothelial dysfunction (Kroller-Schon et al., 2018) that appears to be phagocytic NADPH oxidase (Nox2) (Kroller-Schon et al., 2018) and macrophage/monocyte-dependent (Frenis et al., 2021). These effects can be prevented by induction of the antioxidant principle nuclear factor E2 related factor-2 (Nrf2)/heme oxygenase 1 (HO-1) axis (Bayo Jimenez et al., 2021), implying a critical link between oxidative stress and the onset of adverse cardiovascular effects of noise. This postulate is also supported by numerous oxidative stress markers found in noise-exposed animals such as 3-nitrotyrosine-, malondialdehyde- or 4-hydroxynonenal-positive proteins in different tissues and plasma/serum as well as S-glutathionylated endothelial nitric oxide synthase (eNOS) and uncoupled neuronal nitric oxide synthase (nNOS) and directly measured reactive oxygen species (ROS) formation by high performance liquid chromatography (HPLC)-based quantification of 2-hydroxyethidium and various other staining techniques or oxidative burst (Munzel et al., 2017; Kroller-Schon et al., 2018; Kvandova et al., 2020; Steven et al., 2020; Frenis et al., 2021). Classical biomarkers of oxidative stress such as malondialdehyde-, 4-hydroxynonenal or 3-nitrotyrosine-positive proteins (described for various cardiovascular disease conditions Daiber and Chlopicki, 2020; Daiber et al., 2021) were also observed upon noise exposure.

For a full accounting of the studies of noise exposure in animals with a focus on non-auditory damage see Table 1. However, it is noteworthy that studies arising from different laboratories use different protocols for noise exposure, which accounts for variation in the time of day and duration of the exposure, the length of the noise event (if nonconstant), the type of noise, and the species of the subject.

**TABLE 1:** Studies on non-auditory noise effects on cardiovascular and endothelial dysfunction, inflammation or oxidative stress in animals^a^. Only articles that are not discussed in detail in the main article text are listed here.

Study	Animals and model	Noise scenario	Major outcome of noise exposure	Ref
Peterson 1981	Rhesus Monkey	85 dB, 97 dB peak, unknown type, 9 months	Blood pressure elevation ∼30 mmHg	Peterson et al. (1981)
Borg 1981	Rat	80 dB, 100 dB, unknown type, 10 h, lifelong	Noise-exposed spontaneously hypertensive rats had shorter lifespan and higher incidence of cardiovascular disease, but no differences were found in normotensive rats	Borg and Jarplid (1981)
Peterson 1984	Macaque Monkey	86.6 dB, construction noise, 4 h/8 h, 97 days	Mean blood pressure elevation remained elevated after noise cessation, but heart rate returned to normal relatively quickly	Peterson et al. (1984b)
Kirby 1984	Macaque Monkey	95 dB, broadband noise, 30 m	Offspring of hypertensive monkeys were more sensitive to blood pressure increases from loud noise	Kirby et al. (1984)
Dengerink 1985	Guinea Pigs	120 dB, white noise, 30 m	Effects in cochlear vessel lumen and RBC behavior appear to normalize after 2 days of “noise washout”	Dengerink et al. (1985)
Paparelli 1992	Rat	100 dB, white noise, 12 h	Increased density of noradrenergic cardiac fibers in young animals. In aged animals, increased aortic maximal response to the α-agonist on the aortic musculature and reduced responsiveness to the β-agonist in cardiac fibers	Paparelli et al. (1992)
Morvai 1994	Rat	95 dBA, industrial noise, 6 h, 3 weeks	Noise and alcohol modify the α-adrenergic effect of noradrenaline	Morvai et al. (1994)
Herrmann 1994	Rat	65 dBA, unknown type, 52 weeks	Increased microvessel area, cardiac fibrosis, and ischemic myocardial lesions in SHR exposed to noise	Herrmann et al. (1994)
Breschi 1995	Rat	100 dB, white noise, 1 h/6 h	Diazepam and clonazepam pre-treatment reversed the effects of noise on CBR binding and protected cardiac tissue and aortic responses from the effects of 6 h noise stress	Breschi et al. (1995)
Salvetti 2000	Rat	100 dBA, white noise, 6/12 h	Significant decrease in the binding sites availability of peripheral benzodiazepine receptors following noise	Salvetti et al. (2000)
Singewald 2000	Rat	95 dB, unknown type, 3 m	Noise stress resulted in exaggerated glutaminergic responses in the amygdala of SHR versus Wistar-Kyoto	Singewald et al. (2000)
Bauer 2001	Sheep	161 dB, airborne impulse noise, 20 impulses	Fetal heart rate was affected in both REM and NREM sleep, power of delta, theta, and alpha band power was reduced and cortical activity was detected	Bauer et al. (2001)
Gesi 2002	Mouse	100 dBA, white noise, 6 h	Cardiomyocytes from the right atria and left ventricles display disarranged cristae and matrix dilution in mitochondria	Gesi et al. (2002)
Lenzi 2003	Rat	100 dBA, white noise, 12 h	Increased catecholamine content in myocardium, DNA damage in cardiomyocytes, mitochondrial membrane swelling in right atrium	Lenzi et al. (2003)
Frenzilli 2004	Rat	100 dBA, white noise, 12 h	DNA damage in the adrenal gland, possible redox involvement	Frenzilli et al. (2004)
Baldwin 2007	Rat	90 dB, unknown type, 15 m, 3/5 weeks	Noise increased leakiness of mesenteric arteries, mitigated by vitamin c	Baldwin and Bell (2007)
Antunes 2013	Rat	90 dB, low frequency, unknown duration	Significant myocardial fibrosis detected via CAB staining and alterations in connexin 43 and collagen expression in noise-exposed rats	Antunes et al. (2013a); Antunes et al. (2013b); Antunes et al. (2013c)
Arpornchayanon 2013	Guinea Pigs	106 dB, unknown type, 30 m	TNF-α signaling is activated in the cochlea following noise exposure, causing vessel constriction. Improved by etanercept.	Arpornchayanon et al. (2013)
Gannouni 2013	Rat	70 dB, 80 dB, unknown type, 6 h, 90 days	Increased corticosterone levels, affected various parameters of the endocrine glands and cardiac function. Markers of oxidative stress (catalase, superoxide dismutase and lipid peroxidation) were increased	Gannouni et al. (2013)
Gannouni 2014	Rat	70 dBA, unknown type, 6 h/day, 3/5 m	Structural alterations within the adrenal gland consistent with chronic stress. Signs of necrosis and inflammation in myocardium	Gannouni et al. (2014)
Said 2016	Rat	80–100 dB, chronic and intermittent, unknown type, 8 h, 20 days	Increases in plasma levels of corticosterone, adrenaline, noradrenaline, endothelin-1, nitric oxide and malondialdehyde. Decreases in superoxide dismutase	Said and El-Gohary (2016)
Lyamin 2016	Beluga Whale	140–175 dB, unknown type, 2–4 h, 60 events	Heart rate acceleration following noise exposure. Calves were more susceptible to the effects of noise and did not habituate	Lyamin et al. (2016)
Konkle 2017	Rat	87.3 dBA, unknown type, 15 min–1 h, 21 days	Plasma ACTH, adrenal gland weight, IL6, IL1b levels were unchanged following noise exposure. Increases in TNFα and CRP were seen.	Konkle et al. (2017)
Lousinha 2018	Rat	120 dB, high intensity infrasound, 28 days	Exposed mice had prominent perivascular tissue with notable fibrosis that was mitigated by dexamethasone treatment.	Lousinha et al. (2018)
Yang 2020	Mouse	105 dB SPL, unknown type, 1/4 h	DNA damage response genes appear to fail to respond to noise-induced DNA damage in cochlea, heart, liver, and cortex	Yang and Guthrie (2020)
Lousinha 2020	Rat	120 dB, high intensity infrasound, 12 weeks	Atrial interstitial fibrosis was increased and connexin 43 weas decreased following noise exposure	Lousinha et al. (2020)
Kvandova 2020	Mouse	72 dBA, intermittent aircraft, 4 days	Oxidative parameters and DNA damage increased following noise exposure with synergetic increases in Ogg^-/-^ mice.	Kvandova et al. (2020)
Gogokhia 2021	Rat	High intensity white noise, 1 h, 10 days	Male rats show higher anxiety-like response following noise	Gogokhia et al. (2021)
Bayo Jimenez 2021	Mouse	72 dBA, intermittent aircraft, 4 days	Induction of NRF2/HO-1 protected against oxidative damage, normalized blood pressure, and vascular endothelial function	Bayo Jimenez et al. (2021)

a Table was taken from PhD thesis of Katie Frenis.

## Redox Switches Activated by Noise Exposure

Oxidative stress is a central pathomechanism in response to noise exposure as demonstrated by genetic deletion of the Nox2, which can completely prevent adverse noise effects (Kroller-Schon et al., 2018). We have also demonstrated additive effects of noise-induced oxidative stress with ROS formation originating from angiotensin-II triggered arterial hypertension, an animal model well-known for its pronounced activation of the Nox2 isoform of NADPH oxidases (Steven et al., 2020). Our laboratory also provided molecular proof that the phagocytic Nox2 in lysozyme M (LysM)-positive inflammatory cells (most probably monocytes and macrophages) is responsible for adverse cardiovascular effects of noise since genetic ablation of these LysM-positive cells (by diphtheria toxin treatment of mice with transgenic LysM-specific diphtheria toxin receptor expression) prevented noise-induced vascular oxidative stress, inflammation, endothelial dysfunction and increase in blood pressure (Frenis et al., 2021). A pro-oxidative phenotype was also revealed by RNA sequencing data indicating down-regulation of genes encoding for antioxidant defense proteins such as superoxide dismutase 1 and glutathione peroxidase 1 as well as antioxidant transcription factors such as Forkhead box proteins O (FOXO) (Munzel et al., 2017). Untargeted plasma proteome analysis supported a pro-inflammatory phenotype in noise-exposed mice that was associated with a pro-oxidative shift in ratio of unsaturated to saturated fatty acids, enhanced interaction of leukocytes with the endothelium and overall microvascular dysfunction, which was all corrected by genetic deletion of Nox2 (Eckrich et al., 2021). The noise-induced oxidative stress leads to secondary damage such as adverse redox signaling on eNOS and nNOS as previously reviewed (Daiber et al., 2020). Direct scavenging of nitric oxide by the diffusion-controlled reaction with superoxide also represents a redox switch and supports an antagonistic action of superoxide on nitric oxide signaling (Daiber et al., 2017b).

### Noise Causes Activation of the Phagocytic NADPH Oxidase With Subsequent Redox Activation of Inflammatory Cells

**FIGURE 2:**
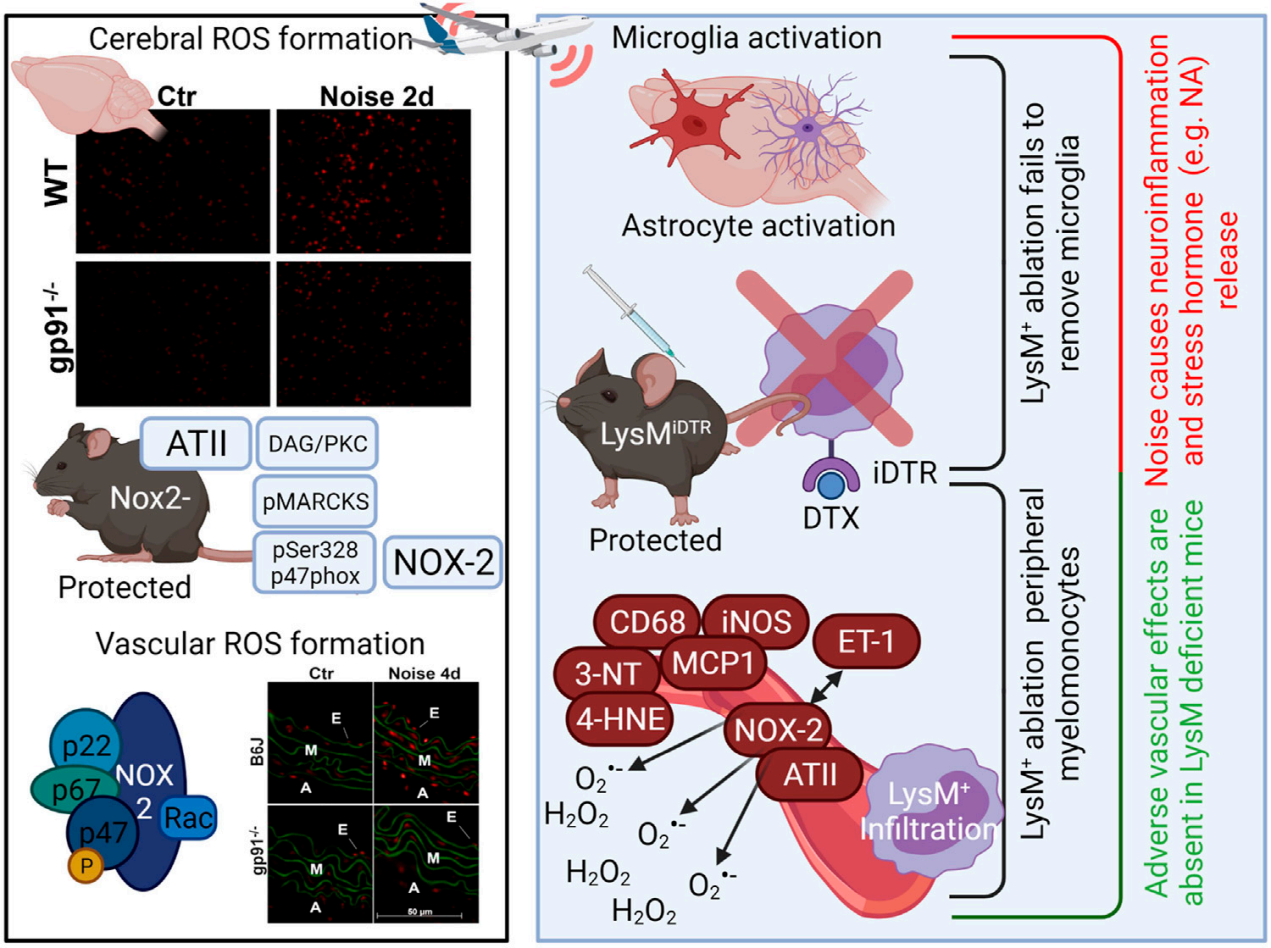
Activation of the phagocytic NADPH oxidase (Nox2, gp91phox) by noise (Kroller-Schon et al., 2018) and role of LysM-positive myelomonocytic cells for noise-induced cardiovascular inflammation and damage (Frenis et al., 2021). Noise causes cerebral and vascular ROS formation as envisaged by more pronounced dihydroethidium (DHE)-derived red fluorescence in cerebral and aortic cryo-sections that was partially corrected in gp91phox (Nox2) knockout mice (representative stainings). Nox2 activation by noise was probably based on angiotensin-II (ATII)-dependent AT1-receptor activation with subsequent activation of phospholipase C and diacylglycerol (DAG) formation, a strong protein kinase C (PKC) activator. PKC activation was documented by noise-triggered phosphorylation of the PKC target myristoylated, alanine-rich C kinase substrate (MARCKS) as well as phosphorylation of p47phox at serine 328, a regulatory cytosolic subunit of Nox2. Translocation of pSer328-p47phox, among other cytosolic regulators, to the cytoplasmatic membrane-bound gp91phox leads to full activation of Nox2 and subsequent superoxide formation. Genetic ablation by treatment of mice with LysM-positive cell (myelomonocytic) specific overexpression of an inducible diphtheria toxin receptor (LysM^iDTR^) with low dose diphtheria toxin (Wenzel et al., 2011). Mice free of LysM-positive cells showed no noise-dependent infiltration of monocytes, macrophages or granulocytes and preserved endothelial function, normal blood pressure and no aortic oxidative stress indicating that LysM-positive cell ablation protects the periphery from noise-induced damage. In contrast, microglia in the brain of LysM^iDTR^ mice were not ablated by diphtheria toxin and noise-induced neuroinflammation, cerebral oxidative stress and release of stress hormones was not prevented. Image was created using Biorender.com. DHE staining images were reused from (Kroller-Schon et al., 2018) with permission.

Professional phagocytes possess a powerful tool to aid in their innate immune activity: Nox2 (or gp91phox). Namely neutrophils, monocytes, macrophages, and their central nervous system (CNS) equivalent microglia are constitutive expressors of Nox2. While this enzyme is critically important in the normal defense against invading pathogens, it also has an apparent role in the development and progression of cardiovascular diseases, including endothelial dysfunction (Chan and Baumbach, 2013), hypertension (Murdoch et al., 2011), ischemic heart disease (Guzik et al., 2006), and atherosclerosis (Sorescu et al., 2002). Importantly, when reconstituting Nox2-containing wildtype monocytes back to LysM-positive cell ablated mice, the protection from angiotensin-II induced hypertension is absent—indicating that vascular impact of Nox2 expression is dominated by its abundance in phagocytic cells (Wenzel et al., 2011). Nox2 inhibition has also been shown to mitigate anxiety-like phenotypes and oxidative stress associated with chronic mild stress (Lv et al., 2019). Accordingly, our own studies demonstrate that upon noise exposure, Nox2 protein and mRNA is consistently upregulated in the murine aorta (Munzel et al., 2017) alongside activation mechanisms of Nox2, such as angiotensin-II dependent diacylglycerol-mediated protein kinase C activation with subsequent phosphorylation of the major cytosolic regulator of Nox2, p47phox, at serine 328 (Figure 2) (Kroller-Schon et al., 2018).

We also find that oxidative stress in the aorta, heart, and brains of noise-exposed mice is significantly increased over those of unexposed controls, which is entirely mitigated in mice with a genetic deletion of Nox2 (Kroller-Schon et al., 2018). These mice were similarly protected from increases in blood pressure, dysregulation of NO signaling, and endothelial dysfunction, which is in line with reports of NOX-derived superoxide being partially determinative in the endothelial dysfunction accompanying genetic, angiotensin, and deoxycorticosterone acetate (DOCA) salt hypertension (Laursen et al., 1997; Zalba et al., 2001; Harrison et al., 2003). Nox2 deletion also protected mice from microvascular dysfunction in the cerebral microvessels and proteomic analysis demonstrated that there was no noise-induced increase in inflammatory signaling in the plasma (Eckrich et al., 2021). In addition, Nox2 inhibition by GSK2795039 suppressed ROS signals in cerebral cryo-sections of noise-exposed mice (Kroller-Schon et al., 2018). It may be also speculated that noise-induced ROS formation promotes an inflammatory phenotype in the heart, vessels and the brain as central mediators of inflammatory reactions such as the NLR family pyrin domain containing 3 (NLRP3) inflammasome and high-mobility group box 1 protein (HMGB1) are activated under oxidative stress conditions via redox switches as well as redox-sensitive transcription factors such as nuclear factor kappa B (NFκB) (Wenzel et al., 2017; Steven et al., 2019). This is probably the reason, aside from stress hormone-dependent activation and infiltration of immune cells into the vasculature, for the observed noise-triggered inflammation in exposed mice (Munzel et al., 2017; Kroller-Schon et al., 2018; Steven et al., 2020; Eckrich et al., 2021; Frenis et al., 2021) but also the shift to a pro-atherothrombotic phenotype of the plasma proteome of train noise-exposed healthy human subjects (Herzog et al., 2019), epigenetic changes that promote immune cell activation and expression of CRP (Cai et al., 2017; Eze et al., 2020) and amygdala activation driven coronary atherosclerosis (Osborne et al., 2020; Hahad et al., 2021a; Osborne et al., 2021). Noise-mediated inflammation in mice was also prevented by genetic Nox2 deletion as shown by two independent preclinical studies (Kroller-Schon et al., 2018; Eckrich et al., 2021) and antioxidant pharmacological activation/induction of the Nrf2-HO1-axis (Bayo Jimenez et al., 2021).

We were able to further discern that Nox2-bearing cells were primarily responsible for noise-induced cardiovascular and cerebral damage through a selective ablation protocol targeting cells expressing lysozyme M. Monocytes and macrophages are generally LysM^+^, whereas microglia are only weakly LysM^+^ or even LysM^−^. As a result, we found that upon ablation, blood pressure, endothelial function, and oxidative stress parameters were largely protected in the periphery (Figure 2) (Frenis et al., 2021). However, an exaggerated stress response measurable through plasma corticosterone level was seen in mice whose monocytes/macrophages were ablated, accompanied by a neuroinflammatory phenotype. Markers of microglial activation, CD68, CD86, and MHC-II, were significantly elevated in flow cytometry analysis of noise-exposed murine brains and not normalized by genetic ablation LysM-positive cells (Figure 2) (Frenis et al., 2021). This apparent disparity somewhat implies that the blood-brain-barrier may be affected by noise, which has been reported in hypertension as well (Setiadi et al., 2018). Furthermore, the pro-oxidative and pro-inflammatory environment appears to have also affected the state of astrocytes in the brains of noise-exposed mice, as an increase in GFAP^+^ staining can be detected. These results are in line with reports of Nox2 activation in microglia in several pathologies affecting the cerebrovasculature (Simpson and Oliver, 2020) and may connect these studies in animals with data from the Gutenberg Health Study of 11,905 participants that demonstrates that annoyance to noise predicts depression and anxiety (Beutel et al., 2020).

### Noise Causes Inactivation and Uncoupling of eNOS

The nitric oxide synthase (NOS) family is critically important for the normal functioning of vessels, due to their role in the production of bioavailable nitric oxide (^•^NO) (Forstermann and Munzel, 2006; Daiber et al., 2019b). Because of the actions of ^•^NO, the presence of normally functioning eNOS and nNOS is cardioprotective (Schulz et al., 2008). However, there is a substantial chink in NOS’s cardioprotective armor: eNOS requires a cofactor, tetrahydrobiopterin (BH4), to facilitate the transfer of electrons in order to produce ^•^NO. The physiological consequence is that when BH4 levels are reduced, the rate at which this electron transfer occurs is slower than the rate of oxidative degradation, which effectively causes NOS to produce superoxide (Forstermann and Munzel, 2006). BH4 can be oxidized to an unusable form by ROS, which sets the stage for NOX-derived ROS to further “kindle” the production of other reactive intermediates by encouraging the uncoupling of NOS enzymes. In fact, superoxide is regarded as somewhat of a direct antagonist of nitric oxide (Gryglewski et al., 1986; Daiber et al., 2017b). Decreased BH4 levels in response to noise exposure were so far not reported.

**FIGURE 3:**
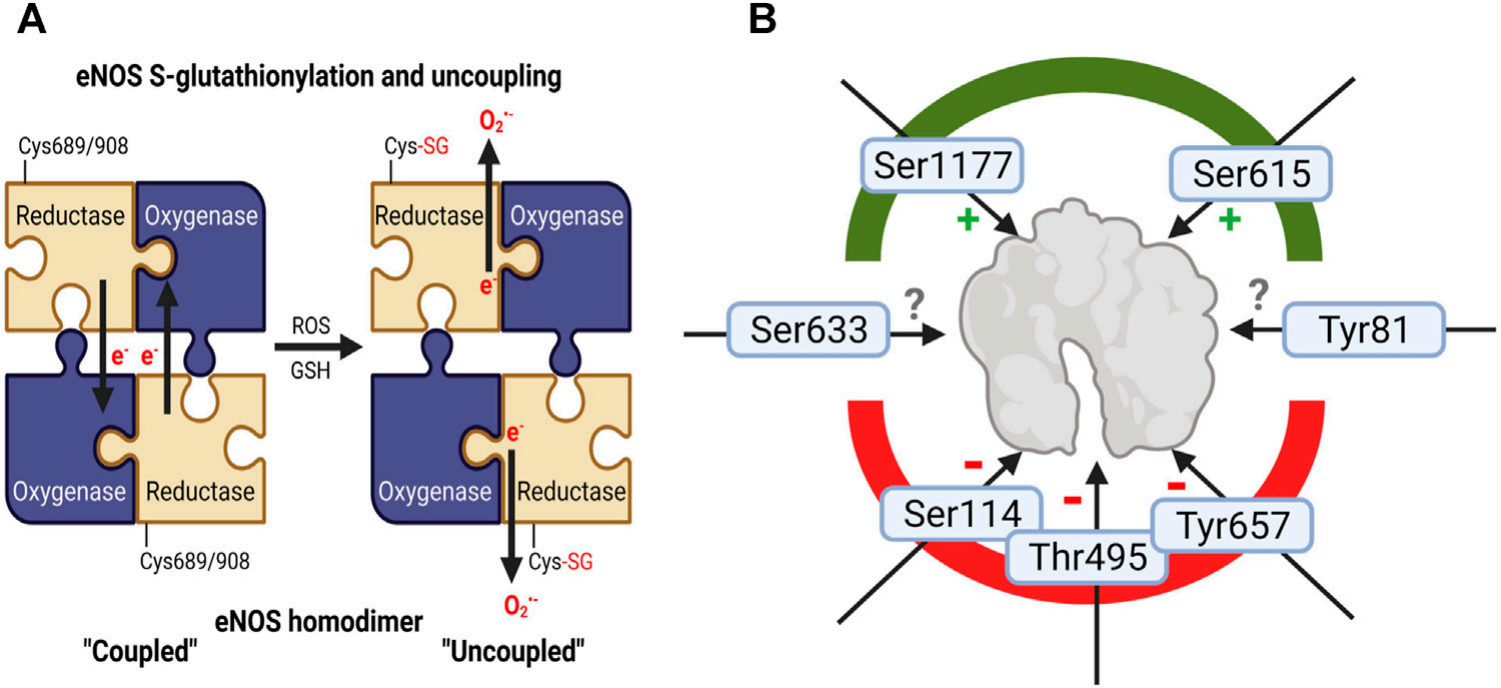
Adverse regulation of eNOS function by noise. **(A)** Schematic explanation of increased eNOS S-glutathionylation in mouse tissues (a surrogate marker for uncoupling of the protein) upon noise exposure (Munzel et al., 2017; Kroller-Schon et al., 2018; Steven et al., 2020). In the “coupled” eNOS homodimer, electrons are usually transferred from the NADPH and flavins to the hem iron. Cysteine residues 689 and/or 908 undergo S-glutathionylation with structural changes (Chen et al., 2010), followed by misdirection of the electrons to molecular oxygen and superoxide formation, termed “uncoupled” state of eNOS. **(B)** eNOS activity is regulated by various kinase-dependent modifications such as activating phosphorylation at serine 1177 or Ser615 and inactivating ones at serine 114, threonine 495 (or 497 depending on the species) and tyrosine 657 (Fleming and Busse, 2003; Mount et al., 2007). Although pThr495-and pTyr657-eNOS was not reported for noise exposure, these inactivating phosphorylations may be expected since they are mediated by oxidatively activated kinases (PKC and PYK-2). Whereas higher eNOS protein expression and Ser1177 phosphorylation was observed in noise (4d)-exposed mice indicating counterregulatory upregulation and activating modification to rescue uncoupling of eNOS enzyme (Munzel et al., 2017; Kroller-Schon et al., 2018), suppression of pSer1177-eNOS was observed in noise-exposed hypertensive mice exposed to 7 days of noise (Steven et al., 2020). Other eNOS phosphorylation sites are not completely explored with respect to their functional effects (Ser633 and Tyr81). Image was created using Biorender.com.

In addition to cofactor BH4 availability, eNOS is tightly regulated through redox mechanisms (Schulz et al., 2014; Daiber et al., 2017a). The redox status of eNOS greatly impacts its synthase activity and can be modulated by the presence of oxidative stress. There are several sites for phosphorylation which can either enhance or decrease the synthase activity of eNOS, however, the most common readouts of eNOS activity are at Ser1177 (Akt-dependent positive effect Dimmeler et al., 1999) as well as Tyr657 and Thr495 (both negative effects) (Figure 3). Importantly, all phosphorylations are redox-sensitive and stimuli-dependent, which is well-established for the protein tyrosine kinase 2 (PYK-2)-dependent phosphorylation at Tyr657 (Fisslthaler et al., 2008; Loot et al., 2009) and the protein kinase C (PKC)-mediated phosphorylation at Thr495 (Fleming et al., 2001; Lin et al., 2003) as both kinases can be activated by hydrogen peroxide. In the presence of oxidative stress, eNOS can also undergo S-glutathionylation, leading to uncoupling (Chen et al., 2010; Karbach et al., 2014). Finally, peroxynitrite appears to have the ability to release zinc from the zinc-thiolate complex coordinating eNOS monomers in the active dimer, representing another mechanism for uncoupling via oxidative stress (Zou et al., 2002).

In our own noise studies, we consistently reported an overexpression and overactivation of NADPH oxidase (NOX) enzymes (Munzel et al., 2017; Kroller-Schon et al., 2018; Steven et al., 2020). It is most likely that due to this overexpression of superoxide-producing enzymes, eNOS in the aorta (and nNOS in the brain) uncouples following noise exposure, as shown by dihydrethidium staining with eNOS inhibitor N^G^-nitro-L-arginine methyl ester (L-NAME) (Kroller-Schon et al., 2018; Steven et al., 2020; Frenis et al., 2021). The paradoxical increase in eNOS protein expression and activating Ser1177 phosphorylation in mice exposed to 4 days of aircraft noise can be best explained by the presence of a largely uncoupled eNOS enzyme. Upregulation of an uncoupled eNOS and increased Ser1177 phosphorylation of an uncoupled eNOS would be detrimental through enhanced superoxide, largely compatible with the observed diminished NO bioavailability (Munzel et al., 2017; Kroller-Schon et al., 2018). However, we also found a reduction in activating phosphorylation at Ser1177 in hypertensive mice who were also exposed to 7 days of noise [mean 72 dB(A)] (Steven et al., 2020). Since oxidative stress was normalized in noise-exposed mice with Nox2 deletion (Kroller-Schon et al., 2018), it is presumptive that extinguishing the initiating spark of superoxide production from Nox2 was sufficient to prevent the uncoupling of eNOS in these mice. In the brains of noise-exposed mice, however, nNOS appeared to be downregulated and uncoupled, which was also preventable through the deletion of Nox2 (Kroller-Schon et al., 2018). In addition, we found eNOS S-glutathionylation in aorta and heart of noise exposed mice (Munzel et al., 2017) that was normalized in Nox2 knockout mice (Kroller-Schon et al., 2018) and was aggravated in an additive manner in noise-exposed hypertensive mice (Steven et al., 2020). Increased eNOS phosphorylation at Thr495 or Tyr657 in response to noise exposure was so far not reported but could be expected due to the redox-sensitivity of the kinases PKC and PYK-2 that confer these phosphorylations. Monomerization of eNOS due to zinc-sulfur complex oxidation in noise-exposed animals was so far also not observed.

### Noise Causes Down-Regulation, Inactivation and Uncoupling of nNOS

In our own studies, noise exposure of mice resulted in decreased nNOS protein expression and triggered uncoupling of nNOS in cerebral tissue. Noise caused phosphorylation of nNOS at serine 847 (Kroller-Schon et al., 2018), which was previously reported to be associated with inhibited (Komeima et al., 2000) or even uncoupled nNOS enzyme (Kasamatsu et al., 2014). Of note, phosphorylation at serine 847 of nNOS is mediated by the redox sensitive calcium/calmodulin-dependent protein kinase (Kasamatsu et al., 2014). The oxidative stress signal in brains of noise-exposed mice could be also partially blocked by specific inhibition of nNOS by ARL-17477, which was in support of nNOS-derived ROS generation and compatible with uncoupling of nNOS enzyme (Kroller-Schon et al., 2018). Oxidative depletion of the protective neurotransmitter ^•^NO also provides an explanation for the observed noise-induced neuroinflammatory phenotype, loss of the protective antioxidant transcription factor Foxo3, all of which contributes to the noise-induced cerebral oxidative stress (Kroller-Schon et al., 2018; Frenis et al., 2021). In addition, suppression of nNOS signaling and shift to a pro-oxidative/inflammatory phenotype of noise-exposed brains provides a feasible explanation for impairment of cognitive development (memory/learning) of school children exposed to high noise levels (Stansfeld et al., 2005). In line with this, impaired learning and memory in adult rats was also found to be associated with Nox2 activity (Kan et al., 2015).

### Noise Upregulates Endothelin-1 That Activates Nox2 and Vice Versa

We also found induction of endothelin-1 expression in the aorta of noise-exposed mice and also exacerbation of endothelin-receptor signaling as envisaged by more pronounced endothelin-1 dependent vasoconstriction (Munzel et al., 2017; Kroller-Schon et al., 2018). Importantly, endothelin-1 is not only one of the most potent endogenous vasoconstrictors but also a potent activator of Nox2 activity, by induction of gene expression (Duerrschmidt et al., 2000; Chen et al., 2012) and direct endothelin-receptor-dependent NADPH oxidase derived ROS formation—demonstrated by *ex vivo* stimulation with endothelin-1 or ROS suppression by ET_A_-receptor blockade of vascular cells (Cerrato et al., 2012; Chen et al., 2012; Steven et al., 2018) or white blood cells (Steven et al., 2017). Endothelin-1 triggered NADPH oxidase-dependent ROS formation was also observed in different models of hypertension (Li et al., 2003a; Li et al., 2003b; Li et al., 2003c). Vice versa, it is also well established that oxidative stress conditions in general and Nox2-derived ROS formation in particular may increase the activity of the endothelin-1 promoter and thereby increase endothelin-1 expression (Kahler et al., 2000; Kahler et al., 2001). Given the cross-activation of Nox2 and endothelin-1, the stimulation of either pathway may lead to a vicious circle that contributes significantly to the cardiovascular oxidative stress and damage (Daiber et al., 2017a). Mitochondrial ROS can also stimulate the release of endothelin-1 as shown in pulmonary artery cells (Ouyang et al., 2012). Endothelin-1 shares also several cross-activation mechanisms with the renin-angiotensin-aldosterone system as evident from higher endothelin-1 expression levels in angiotensin-II treated hypertensive rats (Rajagopalan et al., 1997) and by decreased blood pressure as well as lower plasma angiotensin-II levels in hypertensive animals with bosentan (ET_A/B_ receptor blocker) therapy (Tran et al., 2009). Noise-driven renin-angiotensin-aldosterone system activation by stress hormones can lead to endothelin-1 upregulation or vice versa noise-triggered oxidative stress can stimulate endothelin-1 release and subsequently higher renin-angiotensin-aldosterone system activity. By these mechanisms, endothelin-1 may also contribute to the pronounced toxic effects of noise on Alport (Col4a3^-/-^) mice who display glomerular dysfunction and hearing loss (Meehan et al., 2016).

## Other Noise-Induced Pathways That Affect Systemic Redox Processes or Are Affected by Oxidative Stress

### Noise and the Circadian System

**FIGURE 4:**
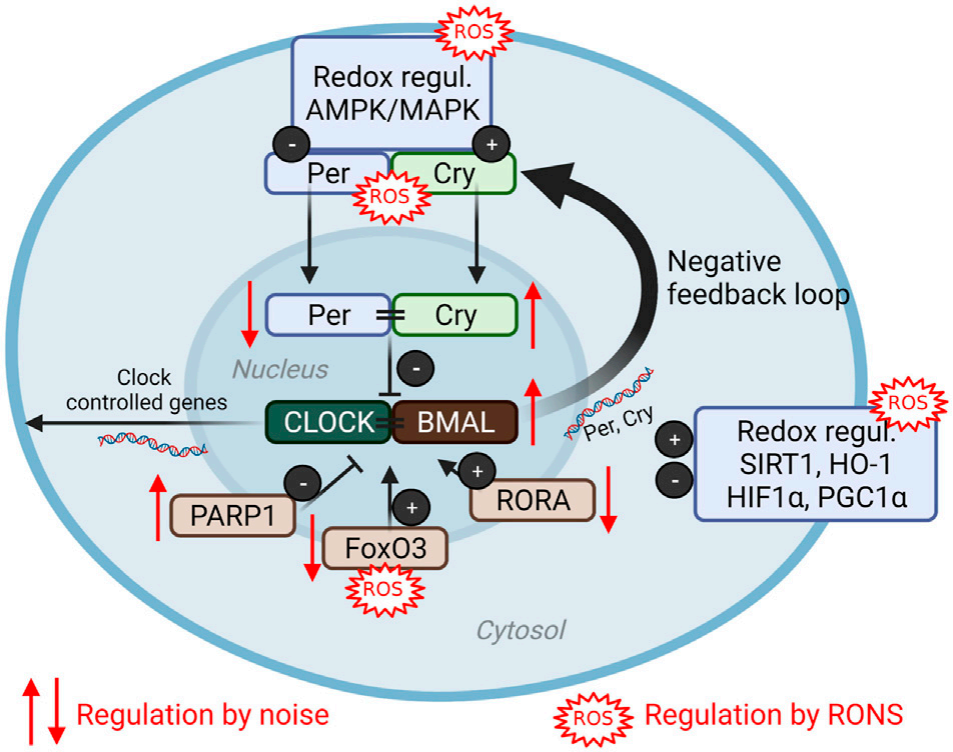
(Redox) dysregulation of circadian clock by noise. The clock core components consist of the positive regulators circadian locomotor output cycles protein kaput (CLOCK) and brain and muscle Arnt-like protein (BMAL) that directly control circadian gene expression as well as the negative regulators period (PER) and cryptochrome (CRY) (Van Laake et al., 2018). Numerous components are redox regulated (reviewed in Li et al., 2020) and modified by aircraft noise exposure of mice (Kroller-Schon et al., 2018). ROS, reactive oxygen species; AMPK, AMP-activated protein kinase; MAPK, mitogen-activated protein kinase; PARP1, poly (ADP-ribose) polymerase-1; FoxO3, forkhead box O; RORA, RAR-Related Orphan Receptor; SIRT1, sirtuin 1; HO-1, heme oxygenase 1, HIF1α, hypoxia-inducible factor 1alpha; PGC1α, peroxisome proliferator-activated receptor gamma coactivator 1-alpha; RONS, reactive oxygen and nitrogen species. Scheme summarized from (Li et al., 2020) with permission under the the terms of the Creative Commons CC BY license. Image was created using Biorender.com.

The circadian clock regulates a number of essential biological functions such as sleep, body temperature, appetite, cognitive functions via time-dependent hormone release such as cortisol or melatonin (Van Laake et al., 2018). Circadian disruption has been identified as a risk factor for cardiovascular disease independently of noise (Crnko et al., 2019), but has also been associated with high (night-time) noise exposure burden (Eze et al., 2017; Munzel et al., 2020; Munzel et al., 2021a) or disrupted sleep pattern such as in shift workers (Furlan et al., 2000; Morris et al., 2016; Thosar et al., 2018). Importantly, given the context of the previous sections detailing the importance of oxidative stress in the adverse effects of noise exposure, redox mechanisms have also been implicated as important in the “redox control of cellular timekeeping” (Putker and O'Neill, 2016). Direct redox modifications of circadian components cryptochrome (CRY), period (PER), and F-box/leucine rich-repeat protein 3 (FBXL3) arise as thiol oxidation/reduction and the formation or disruption of zinc-sulfur complexes, which then control the binding of these components to the regulators circadian locomotor output cycles protein kaput (CLOCK) and brain and muscle Arnt-like protein 1 (BMAL1) complex, an essential part of the feedback mechanism inherent to circadian control (Figure 4) (Schmalen et al., 2014). While the direct redox modifications of clock components in the context of noise exposure have yet to be realized, other regulatory redox mechanisms also exist. Redox-sensitive kinases, histone deacteylases, stress-response proteins, and transcription factors can be modulated by the presence of ROS with further impact on the clock system (Figure 4) (Li et al., 2019a). The impact of various environmental stressors, including mental/social isolation stress, air pollution, heavy metals and pesticides on the circadian clock, especially its adverse redox regulation, was reviewed in (Li et al., 2020).

Bridging the concepts of circadian disruption by noise and redox control of the clock system, there is evidence of an important role in environmental cues and stressors in the regulation of the circadian rhythm (Li et al., 2019a). In mice exposed to continuous aircraft noise for 4 days [mean sound pressure level (SPL) of 72 dB(A)], expression patterns of key components of the circadian pathway in aorta and kidney were altered in comparison to unexposed controls (Kroller-Schon et al., 2018), including downregulation of Per1 and REV-ERB-α/β (Nr1d1/2) or RORα and upregulation of Bmal1, Cry1, Cul1, Prkag1/2, Parp1. In total, more than 30 circadian genes were altered in their expression levels. Downregulation of forkhead-box-protein O3 (FoxO3), a transcription factor that seemed to function as a central signalling hub regulating the circadian genes in the vascular tissue, was also reported. Pharmacological activation of FoxO3 by bepridil successfully prevented noise-induced oxidative stress in the aorta and the endothelial dysfunction that arises from it (Kroller-Schon et al., 2018). In a study of the transcriptomics of neurons within the inferior colliculus, a brain structure that has an important role in sound processing, distinct profiles between day and night-time exposure appeared in clock genes (Park et al., 2016).

### Noise and the Microbiome

**FIGURE 5:**
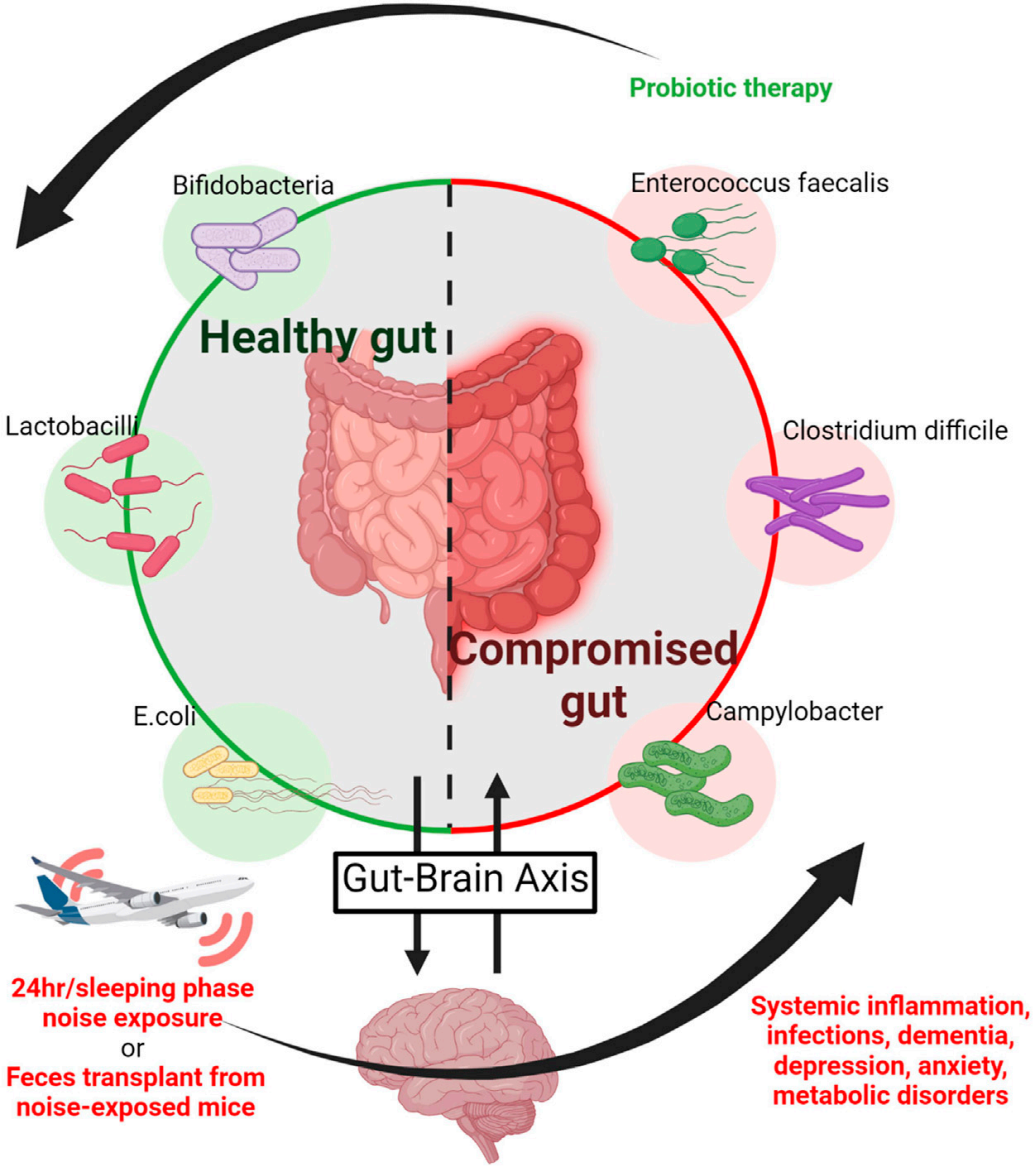
Noise and the microbiome. The gastro-intestinal microbiome is connected to neuropsychiatric processes via the gut-brain axis and thereby affects neuropsychiatric disorders, whereas mood and neuropsychiatric health may affect intestinal inflammatory disease (Collins et al., 2012; Cryan and Dinan, 2012). Noise causes neuronal activation with subsequent stress hormone release and is associated with annoyance, depression and dementia. Accordingly, noise triggers alterations of the gut-brain axis leading to a shift to harmful bacteria in the intestine associated with cognitive impairment and Aβ accumulation in a murine model of Alzheimer’s disease (Cui et al., 2018). Noise also disrupts the equilibrium of intestinal pro-oxidative and antioxidant mechanisms in association with low-grade systemic inflammation in mice (Chi et al., 2021) and generally causes an imbalance of health-compromising versus -promoting bacteria together with impaired mental health. As a proof-of-concept these adverse health effects of noise where mostly corrected by probiotic therapy (Hadizadeh et al., 2019), whereas feces transplantation from noise-exposed to unexposed mice induced the above mentioned health complications (Cui et al., 2018). Image was created using Biorender.com by modifying the central scheme from https://de.freepik.com/vektoren-premium/menschlicher-doppelpunktvektor-der-guten-bacterial-flora-illustration_3804027.htm.

The investigation of gut microbiota in the pathomechanisms of disease has experienced an explosion in recent years. The microbiome affects fundamental processes such as inflammation and redox signalling in the gastro-intestinal tract (Figure 5) (Campbell and Colgan, 2019). As a result, significant associations between the state of gut microbiota and cardiometabolic diseases have been made (Jones and Neish, 2017; Campbell and Colgan, 2019). Additionally, the existence of a gut-brain axis appears to be a central player for mood and behavior regulation as well as for the development of neuropsychiatric disorders and intestinal inflammatory disease (Collins et al., 2012; Cryan and Dinan, 2012). This may also be of particular interest for the present review as transportation noise is obviously also associated with a higher incidence of all-cause dementia, namely Alzheimer’s disease (Cantuaria et al., 2021). These states are noteworthy in the current context due to their known cardiovascular and mental risk (Hahad et al., 2019). Relatively few studies explicitly probe the relationship between noise exposure and alterations of the gut microbiome, but those that have been conducted show notable effects of noise. In one study of chronic exposure for 4 h/d during the sleeping phase over the course of 30 days [88–98 dB(A)], alterations of the microbiome-gut-brain axis were reported (Cui et al., 2018). The mice of the study were a model for Alzheimer’s disease, and chronic noise exposure was associated with cognitive impairment and amyloid beta peptide (Aβ) accumulation. The mice correspondingly had decreased neurotransmitter levels (5-HT and GABA), increased markers of neuroinflammation, and impaired intestinal and brain endothelial tight junction protein expression (e.g., claudins and occludin). Underlying these changes, alterations of the intestinal flora were revealed by 16S ribosomal RNA sequencing, which was supported by additional experiments utilizing fecal transplantation (Figure 5). Feces from mice exposed to 98 dB(A) noise were transplanted into unexposed mice, who subsequently developed an Alzheimer-like phenotype (Cui et al., 2018). Changes in the gut microbiome in a mouse model for Alzheimer’s disease were associated with an imbalance between intestinal pro-oxidative and antioxidant pathways as well as low-grade systemic inflammation in response to noise exposure (Chi et al., 2021).

Results of altered gut composition were also reported for noise-exposed rats in a similar experimental exposure (Cui et al., 2016), as well as an alteration in the balance of health-compromising proteobacteria and health-promoting actinobacteria as measured by 16S rRNAseq (Zymantiene et al., 2017). These noise-induced changes in microbial balance were accompanied by increased TNF-α and IL-1β as well as alterations of body weight, and haematological parameters as well as histopathological changes in the organs (Zymantiene et al., 2017). Anxiety-like behavior arose following noise exposure in rats, where higher serum corticosterone levels reflecting the increased stress response. Probiotic treatment alleviated these symptoms by apparently restoring the gut-brain axis (Figure 5) (Hadizadeh et al., 2019). Though the data up until now is rather sparse, these early findings indicate that noise disruption of the gut-brain axis through disturbance of the gut microbiota could be exacerbating the inflammatory phenotype that arises following noise exposure, which could potentially lead to cardiometabolic disease development (Karl et al., 2018).

### Noise and Metabolic Syndrome

Metabolic syndrome comprises a cluster of co-occurring conditions which lead to or complicate cardiometabolic disease. These conditions include hypertension, hyperglycemia, insulin resistance, dyslipidemia, type 2 diabetes, nonalcoholic fatty liver disease, and dementia. These conditions are all associated with oxidative stress via the increased production of ROS (Spahis et al., 2017a; Spahis et al., 2017b; Carrier, 2017). Notably, metabolic syndrome has known associations with the two modes of disturbance outlined in the noise reaction scheme: sleep and stress. Metabolic syndrome has a positive correlation with both abnormal sleep patterns, such as overly long or short duration (Smiley et al., 2019), sleep apnea (Borel, 2019), as well as circadian disruptions (Depner et al., 2014).

While we are unaware of any translational animal studies explicitly investigating metabolic syndrome as a cluster, there are several studies in mice and rats that focus on type 2 diabetes and insulin resistance. One such study found that diabetes induced by high fat diet was worsened by merely 4 h/d of 85 dB SPL noise exposure, as measured through glucose intolerance, insulin resistance, fasting hyperglycemia, and apparent dyslipidemia (Liu et al., 2018b). Another study found that in male mice, 4 h/d of 95 dB SPL noise exposure caused insulin resistance accompanied by phosphorylation of Akt, IRS1, and JNK, increased levels of circulating inflammatory cytokines TNF-α and IL6, and increased SOD and catalase activity, indicative of oxidative stress (Liu et al., 2018a). Insulin resistance was also documented in noise exposure of 1, 10, and 20 days (Liu et al., 2016). Rats exposed to 28 days of 95 dB noise were also found to have increased corticosterone, triglycerides, total cholesterol, and altered the balance of lipoproteins (Morakinyo et al., 2019). These findings are also in agreement with observational studies on the prevalence and incidence of metabolic syndrome conducted in humans, both during occupational and other noise exposures (Huang et al., 2020; Khosravipour et al., 2020; Yu et al., 2020). Road traffic noise was also associated with incident diabetes in the population-based Danish Diet, Cancer and Health cohort comprising 57,053 participants (Sorensen et al., 2013). Overall, there is evidence of a possible link between high noise exposure and several components of metabolic syndrome, with a possible mechanistic link through stress and sleep disruption prompting the production of ROS, though more investigation is certainly required.

### Noise and Epigenetic Pathways

Epigenetic changes can modulate the development and progression as well as the severity of cardiovascular diseases by control of atherosclerotic processes (Ordovas and Smith, 2010; Kuznetsova et al., 2020). Epigenetic processes are largely redox-regulated (Mikhed et al., 2015; Kietzmann et al., 2017; Leisegang et al., 2017) and thereby noise-induced oxidative stress will most likely change the epigenetic landscape at multiple layers. We and others reported noise-induced changes of coding RNA by next-generation sequencing in models of non-auditory noise effects (Munzel et al., 2017; Kroller-Schon et al., 2018) and studies on hearing loss (Wei et al., 2020; Lavinsky et al., 2021). However, noise exposure, sleep deprivation and mental stress can also lead to altered expression patterns of non-coding RNA, e.g., in microRNAs that have significant health impact (Miguel et al., 2018; Miguel et al., 2020). The dysregulation of microRNAs can be mediated by the indirect pathway, e.g., the known stress response, but also via direct mechanical damage of the inner ear during hearing loss (Miguel et al., 2018). Higher expression levels of miR-134/183 in the central amygdala were observed after acute stress exposure (Meerson et al., 2010). Both microRNAs seem to have significant health impact as they were found at higher concentrations in patients with coronary artery disease and depression. Numerous of these microRNAs that are associated with environmental risk factors such as noise exposure or mental stress are either regulated by oxidative stress or themselves influence gene transcription encoding for antioxidant defense or pro-oxidative proteins (Miguel et al., 2018; Miguel et al., 2020). Methylation of DNA bases is another epigenetic regulatory process with large impact on cardiovascular risk (Greco and Condorelli, 2015). Alterations of the DNA methylome, the sum of all methylated DNA bases with significant effect on transcriptional activity of DNA, were demonstrated in the brain of rats after chronic noise exposure, pointing towards epigenetic regulation of metabolic pathways by stress signalling in the form of noise exposure (Guo et al., 2017). Of note, a cohort study (SAPALDIA) conducted in Switzerland found an association between long-term exposure to transportation noise and DNA methylation patterns indicating activation of inflammatory pathways, alterations of cellular development and changes of immune responses (Eze et al., 2020). Epigenetic effects observed by human and animal studies on hearing loss but also epigenetic changes in non-auditory models were reviewed in (Leso et al., 2020).

## Conclusion

In conclusion, noise is a somewhat “pleiotropic” stressor, with the ability to incur damage through both cognitive and noncognitive input pathways. Cognition of noise, as happens during noise exposure while awake, appears to be linked to the anxiety and depression, as reported following noise exposure in humans. These symptoms in humans correspond well with a neuroinflammatory phenotype stemming from both astrocytic and microglial activation in mice, accompanied by downregulation and uncoupling of nNOS. Critically, noise exposure also activates both the SNS and HPA axis, causing hormonal dysregulation, which can inflict changes in the peripheral systems. Studies in mice suggest that these hormonal disruptions coupled with circadian interruption promote the production of oxidative stress, which appears to be the common thread through all the detrimental effects of noise and seems to be largely based on Nox2 activation as the major source of ROS. In short, infiltration of monocytes and macrophages in response to stress appears to trigger the production of oxidative stress, which then uncouples e/nNOS via specific redox switches, disrupts nitric oxide signaling, disturbs essential phosphorylation within circadian pathways, and activates ROS-sensitive transcription factor NFκB as well as defense systems such as Nrf2/HO-1 or causes impairment of FoxO3 signaling. These effects also have potential for affecting epigenetic regulation and microbiome homeostasis. Because of the importance of these effects for affecting human health, it is necessary for noise research to be conducted in a systemized manner in both humans and animals to explore both the unknowns in redox and cardiovascular biology, but also those in other fields.

## Future Directions

The molecular underpinnings of noise-induced physiological consequences appear as a consequence of hormonally-induced hyperactivity of cells of the monocytic line bearing Nox2. Cellular metabolic changes are not only important in cardiovascular research, but also in several other fields of study, including cancer and neurological disorders. Given that the effects in the brain are so notable in translational work and behavioral and emotional effects are apparent in humans, there appears to be a wide field of study in the effects of noise within both the cerebrovasculature and in directly studying neuronal health. Since mice have thus far been a relatively faithful model for at least one mode of noise exposure, translational studies investigating the behavioral and cognitive effects of noise are warranted. Our own work suggests that there is a combinatorial effect between pre-existing hypertension and noise exposure, which worsens the phenotype. Additional study into the effects of noise in other disease states appears to have potential for linking the exposome to tangible effects on human health.

## Limitations

Though the field of noise research is quickly expanding, the major limitation remains to be a paucity of mechanistic studies. Most of the investigations in humans are through the lens of occupational exposure to noise, which is often high-intensity and acute, whereas the majority of people are exposed in lower levels in their daily life (i.e., through ambient traffic noise). While the consensus that very high exposure has links to metabolic and cardiovascular consequences, more and better standardized studies are required to investigate the everyday noise burden, especially at a mechanistic level on-top of the so far mostly observational epidemiological studies that focus on the overall health impact (e.g., disease incidence and prevalence). Another significant limitation is the variance in exposure: each individual’s daily exposure will vary, which complicates observational studies in human communities. Though mouse models can replicate the consequences of noise in some aspects, it is unlikely that mice can feel the depth of emotional response a human would to an unwelcome noise, meaning that translational research can only reflect one half of the noise-reaction scheme and that the noise in these experiments is probably imparting its effects through sleep disruption. This may be overcome by technical advances in the field of personal monitoring devices, which would allow continuous recording of the noise exposure levels of the individual during daily life. Lastly, it is notable that the majority of translational studies are conducted in male mice, which is useful for lower variation range of the data but may not accurately reflect the range of response to a stressor such as noise that is largely affected by alterations of hormonal pathways that are known to show significant differences between males and females.

## Abbreviations

Aβ, amyloid beta; ACTH, adrenocorticotropic hormone; BMAL1, brain and muscle arnt-like protein; BH4, tetrahydrobiopterin; CLOCK, circadian locomotor output cycles kaput; CRP, C-reactive protein; CRY, cryptochrome; d, day; dB, decibel; DHE, dihydroethidium; eNOS, endothelial NOS; FOXO, forkhead box O; FBXL3, F-Box and leucine rich repeat protein 3; h, hour; HPA, hypothalamic-pituitary-adrenal; HO-1, heme oxygenase-1; IL, interleukin; LysM, lysozyme M; NFκB, nuclear factor kappa-light-chain-enhancer of activated B cells; nNOS, neuronal NOS; NOS, nitric oxide synthase; Nox2, NADPH oxidase 2; Nrf2, nuclear factor-erythroid factor 2; PKC, protein kinase C, PYK-2, protein tyrosine kinase 2; ROS, reactive oxygen species; SNS, sympathetic nervous system; SPL, sound pressure level; TNFα, tumor necrosis factor alpha; WHO, world health organization.
